# Effects of dehorning on population productivity in four Namibia sub-populations of black rhinoceros (*Diceros bicornis bicornis*)

**DOI:** 10.1007/s10344-022-01607-5

**Published:** 2022-08-15

**Authors:** Lucy C. Chimes, Piet Beytell, Jeff R. Muntifering, Birgit Kötting, Vikki Neville

**Affiliations:** 1 Bristol Veterinary School, University of Bristol, Langford, Bristol, BS40 5DU UK; 2 Ministry of Environment, Forestry and Tourism, P/Bag 13306, Windhoek, Namibia; 3 Save the Rhino Trust, PO Box 2159, Swakopmund, Namibia; 4 Great Plains Zoo, 805 S. Kiwanis Avenue, Sioux Falls, SD USA; 5 Namibia University of Science and Technology, Private Bag 13388, Windhoek, Namibia

**Keywords:** Poaching, Black rhinoceros, Namibia, Dehorn, Population productivity

## Abstract

The black rhinoceros (rhino) (*Diceros bicornis*) is critically endangered, with poaching being one of several threats to the species’ survival. Many reserves across several countries, including Namibia, South Africa, and Zimbabwe, now dehorn their rhinos in an attempt to reduce poaching. Historical data collected by the Namibian Ministry of Environment, Forestry, and Tourism and Save the Rhino Trust were used to investigate whether dehorning has an effect on age at first reproduction (AFR), inter-calving interval (ICI), birth sex ratios, calf survival, cause of death, and lifespan. These were assessed in four Namibian sub-populations (hereafter referred to as ‘populations’) of black rhino (denoted A, B, C, and D) which have undergone varying levels of dehorning. No significant difference was found in any of the variables between dehorned and horned individuals. Population was a significant predictor of AFR (LRT = 7.433, *p* = 0.024) and ICI (LRT = 13.281, *p* = 0.004), although pairwise comparisons only found populations A and B to be significantly different (AFR: *z* = −2.736, *p* = 0.017, ICI: *z* = −3.705, *p* = 0.001). Additionally, a significantly higher number of males than females were born in population D (*p* = 0.021, CI = 0.544, 0.960). The main cause of death across all individuals was poaching, although there was no significant difference in the proportion of rhinos poached between dehorned and horned individuals (*X*^2^ = 0.638, *p* = 0.424, *n* = 265). No evidence was found to suggest that dehorning has any effect on AFR, ICI, birth sex ratios, calf survival, or lifespan in the black rhino, which is reassuring in the continued use of dehorning as an anti-poaching technique in this species.

## Introduction

Rhinoceros (rhino) species, and their parts and derivatives, accounted for 11.8% of illegal wildlife seizures by value between 2014 and 2018, the third highest animal group behind elephants and pangolins (UNODC 2020), with trade primarily driven by demand for rhino horn in traditional medicine and as a status symbol (Milliken and Shaw 2012; Truong et al. 2016). All five extant rhino species are listed in Appendix I of the Convention on International Trade in Endangered Species of Wild Fauna and Flora (CITES), with the exception of the South African and Eswatini populations of the subspecies *Ceratotherium simum simum*, which are listed in Appendix II. As described in Article III of CITES, Appendix I listing prohibits international commercial trade in specimens of these species, including rhino horn. This means that demand for rhino horns is being met illegally, driving a poaching crisis, primarily across southern Africa.

Two species of rhino are endemic to Africa: *Diceros bicornis* (black rhino) and *Ceratotherium simum* (white rhino). High levels of poaching, particularly in southern Africa, are putting both of these species at increased risk of extinction despite the implementation of extensive anti-poaching strategies (Lindsey and Taylor 2011; Cheteni 2014; Mukwazvure and Magadza 2014; Emslie et al. 2019; Haas and Ferreira 2016; Crookes and Blignaut 2019; Ferreira et al. 2015). They are currently listed as critically endangered (Emslie 2020b) and near threatened (Emslie 2020a), respectively, on the International Union for Conservation of Nature’s Red List (IUCN Red List). For this reason, some national parks and reserves across several countries, including South Africa, Namibia, and Zimbabwe, have resorted to dehorning in an attempt to decrease poaching pressure (Lindsey and Taylor 2011). Dehorning is the controlled removal of the majority of the rhino’s horn (Kock and Atkinson 1993), which decreases the weight and therefore value of horn remaining, reducing poaching incentives (Lemieux and Corné 2014).

It has been suggested that dehorning has some effect on rhino behaviour and biology, either through the possible consequences of having no horn or the dehorning process itself, which requires chemical immobilisation. Dehorning may also have an indirect stress effect through disturbance caused by the interventions required for dehorning, including vehicles, helicopters, and capture teams. The potential effects of dehorning have been investigated, with studies reporting its impact on a range of factors including calf survival (Berger and Cunningham 1994; Atkinson 1996; Du Toit and Anderson 2013), inter-calving interval (ICI) (Alibhai et al. 2001; Atkinson et al. 2002; Du Toit 2001; Penny et al. 2020a), and corticosteroid levels (Badenhorst et al. 2016; Penny et al. 2020b). Dehorning was found to have no significant long-term effect on corticosteroid levels in two studies on white rhinos (Badenhorst et al. 2016; Penny et al. 2020b). However, some of the studies show conflicting results. Berger and Cunningham (1994) reported 100% calf mortality in the offspring of dehorned mothers, whilst Atkinson (1996) and Du Toit and Anderson (2013) report have observed no difference in calf survival between dehorned and horned mothers. Serious questions have since been raised about Berger and Cunningham’s methodology (Lindeque and Erb 1995). Meanwhile, a study by Alibhai et al. (2001) voiced concern that the immobilisation of rhinos (crucial for dehorning) can result in increased ICIs, whereas Penny et al. (2020a) reported decreased ICIs in dehorned individuals. Several of these studies have concentrated on the white rhino, and therefore the results of the current study will provide a valuable addition to the available literature on the dehorning of black rhinos. Further research is needed to better understand the consequences of dehorning and to provide better support to the belief that dehorning itself is not detrimental to the population growth of this critically endangered species, as this could further undermine their long-term survival.

Namibia is home to one of the world’s largest remaining populations of black rhino (t’ Sas-Rolfes 2016), accounting for an estimated 34% of the global population, and in particular, 85% of the world’s population of the *Diceros bicornis bicornis* subspecies of black rhino (Emslie et al. 2019). Dehorning began here in 1989, carried out by the Namibian Ministry of Environment, Forestry and Tourism (MEFT), making it the first country to use dehorning as an anti-poaching method (Lindsey and Taylor 2011). Dehorning was terminated in Namibia in 1995 but restarted in 2014 due to rising poaching levels and has been carried out regularly ever since (MEFT, personal communication 2021). Population productivity is important to the survival of these populations, and so it is vital that this is not impacted by dehorning. Population productivity has been widely assessed in the rhino previously through a range of factors, including calf survival, age at first reproduction (AFR), and ICI (Law et al. 2013; Alibhai et al. 2001; Penny et al. 2020a; Hrabar and Du Toit 2005; Freeman et al. 2014).

The aim of this study was to use data collected by the MEFT and Save the Rhino Trust (SRT) to compare population productivity between dehorned and horned individuals. Four sub-populations (hereafter referred to as ‘populations’) were assessed, including three which have been exposed to varying levels of dehorning, and one control, which has never been dehorned. It was hypothesised that dehorning would not have a detrimental effect on population productivity. In particular, it was predicted that there would be no significant difference in AFR, ICI, cause of death, birth sex ratios, or calf survival between dehorned and horned individuals. Lifespan was expected to be higher in dehorned than horned individuals due to the use of dehorning as an anti-poaching method (Lindsey and Taylor 2011). One of the study populations (population A) is found in a desert-like environment and is exposed to harsher climatic conditions, so these individuals are likely to be more physiologically stressed. Therefore, it was anticipated that there would be differences in population productivity between this group and the other study populations. It was also predicted that should dehorning have an impact on the variables assessed here, it would be most apparent in this population (A).

## Methods

### Ethics

This research was approved by the University of Bristol Animal Welfare and Ethical Review Body (reference number: UIN/21/049) and the Namibian National Commission on Research, Science and Technology (permit number: RPIV01042026).

### Study populations

This study assessed four Namibian populations of the *Diceros bicornis bicornis* subspecies of black rhino using data collected by the MEFT and SRT since 1973 for population A, and since the introduction of rhinos to the sites in populations B (2000), C (1996), and D (2008). The populations have been exposed to different environmental stresses and management techniques (Table 1), allowing for comparison at the population level and also between dehorned and horned individuals in varying conditions. Individuals were defined as dehorned if they had been dehorned at least once. The populations are referred to as A, B, C, and D. Population A is a free-ranging population outside of a formally protected area, whilst Populations B, C, and D are part of the Black Rhino Custodianship Programme, which provides additional security. Dehorning has taken place regularly in populations A, C, and D since 2014, whilst population B has never been dehorned.

**Table 1:** Comparisons between the study populations’ habitats and climatic conditions

**Population**	**Area (km**^**2**^**)**	**Average annual rainfall (mm)**	**Diurnal temperature range (°C)**	**Habitat type**	**Management**	**Dehorning status**
A	290	150 (Brodie et al. 2011)	Winter: 5–26 Summer: 14–35 (Inman et al. 2020)	Basalt and schist foothills with mountains and gravel plains (Brodie et al. 2011). Sparse vegetation is dominated by dwarf shrubs and annual grass species (Namibian Ministry of Environment and Tourism 2010)	The free-ranging population outside of formally protected areas (Brodie et al. 2011). Part of a communal custodianship	Every 2 years since 2014. 36.8% of individuals were dehorned at least once across the study period
B	300	351 (Matson et al. 2006)	Winter: 6–25 Summer: 18–35 (Gasaway et al. 1996)	Mixed shrubland on calcrete with rocky outcrops (Matson et al. 2006). Dominant vegetation is dwarf shrubs and annual grass species (Namibian Ministry of Environment and Tourism 2010)	Private game reserve with a focus on wildlife conservation. Part of the Black Rhino Commercial Custodianship Programme	No dehorning
C	70	250–400 (Namibian Ministry of Environment and Tourism 2010)	Winter: 4–25 Summer: 12–32 (Mendelson et al. 2002)	Located on the border of the Kalahari. Variety of grass and acacia species, including camelthorn and blackthorn (Namibian Ministry of Environment and Tourism 2010)	Game camp as part of a free-hold farm. Part of the Black Rhino Commercial Custodianship Programme	Every 3 years since 2017. 48.2% of individuals were dehorned at least once across the study period
D	96	450–700 (Mendelsohn and El Obeid 2002)	Winter: 3–21 Summer: 12–30 (Mendelsohn and El Obeid 2002)	Terrain is mainly Kalahari sand (Mendelsohn and El Obeid 2002). High species diversity with deciduous tree species including Zambezi teak, mopane and wild seringa (Namibian Ministry of Environment and Tourism 2010)	Double-fence with electric fence. Part of the Black Rhino Commercial Custodianship Programme, co-managed by the conservancy and the MEFT (communal custodian)	Every 2 years since 2014. 81.8% of individuals were dehorned at least once across the study period

### Measures of population productivity

The key outcome measures, each relevant to population productivity, were: calf survival, ICI, AFR, birth sex ratios, lifespan, and cause of death. Calf survival was defined as survival to the sub-adult life stage when the calf is no longer dependent upon its mother. The transition from calf to sub-adult occurs at the first of the following; separation from the mother, the birth of the subsequent calf, or the calf’s fourth birthday (Emslie et al. 1995; Law et al. 2013). Percentage calf survival was calculated for each female, with only native calves included in the analysis. Native calves were defined as those which were born in the study population rather than having been translocated into the population. Calves for which it was unknown whether their mother had been dehorned were excluded from dehorning analysis. Mothers were categorised as dehorned if the procedure occurred during the time that the calf was dependent on the mother or if the dehorning occurred in the 3 years prior to the birth of the calf. This accounted for the approximately 2 years required for horn regrowth (Lindsey and Taylor 2011) and an additional buffer year to allow for variation in individual regrowth rates.

ICI and AFR were used as measures of reproductive success because they are not skewed by poaching. Due to the potential influence of translocation, only native mothers were included in the AFR analysis. Native mothers were defined as those who were born in the study population rather than having been translocated into the population. Population D was not included in the AFR analysis because to date, no native females have given birth in this group. Introduced individuals were included in ICI analysis, although ICIs prior to introduction and covering the translocation period were excluded. Mean ICI for each population was calculated as the average mean ICI per female. ICIs spanning the dehorning event were not included in pre- and post-dehorning comparisons because ICI length could not be fully attributed to dehorning as some dehorning took place late in the ICI.

Birth sex ratios were calculated as the total number of male and female calves born in each population with unsexed calves and calves born prior to introduction excluded from analysis.

Mortality was assessed through lifespan and cause of death. Lifespan was calculated as the estimated age at death of deceased individuals where deaths had occurred in the populations within the study timeframe. Lifespan was compared both across populations and between dehorned and horned individuals. The impact of the number of times an individual had been dehorned on lifespan was also assessed. The cause of death was categorised into six groups: fighting, natural causes, poaching, predation, other and unknown. Deaths caused indirectly by these categories, for example, individuals which were euthanised due to poaching-related injuries, were attributed to these groups based on the primary cause (e.g. poaching). Individuals were categorised as dehorned at the time of death if they had been dehorned within 3 years of the recorded death date to account for approximately 2 years required for horn regrowth (Lindsey and Taylor 2011) and an additional year to accommodate variation in regrowth rates between individuals.

To correct for data falling outside of the study period, for the analysis of AFR, birth sex ratios, calf survival, and cause of calf death, only data from individuals born in the populations was included, meaning that all data for these variables fell within the study period. For ICI analysis, only ICIs recorded within the populations within the study period were assessed. Due to small sample size, individuals translocated into the populations were included in lifespan and cause of death analysis.

### Statistical analysis

All statistical analysis and data presentation were carried out using RStudio Version 1.4.1717 (RStudio Team 2021).

General linear models (GLM) implemented in the R base package were used to test whether dehorning (dehorned and horned) or population (A, B, C, and D) were significant predictors of variation in lifespan (years), to test whether the number of times that an individual was dehorned is a significant predictor of lifespan; and to test whether population (A, B, C, and D) was a significant predictor of mean calf survival per female (percentage), mean AFR (years), or mean ICI (months). A binomial GLM was used to assess whether dehorning (dehorned and horned) of the mother was a significant predictor of calf survival (binomial, 1 = survival to sub-adult, and 0 = died prior to the sub-adult stage). In GLMs comparing variables between dehorned and horned individuals, the population was included in the model as a covariate.

Lifespan and mean calf survival per female data were log-transformed to ensure that the assumptions of homogeneity of variance and normality of error were not violated. Likelihood ratio tests (LRT) were used to assess the significance of predictor variables in the GLMs, and pairwise comparisons between populations were made using Tukey’s honestly significant difference (HSD) test implemented in the multcomp package (Hothorn et al. 2008).

Binomial tests (R base package) were used to compare birth sex ratios between populations (number of male births per population compared to the total number of known-sex calves per population) and before and after dehorning within populations (number of male births prior to the mother being dehorned compared to the number of male births after the mother was dehorned within the same population).

*X*^2^ tests (R base package) were used to assess whether there was a significant difference between the proportion of dehorned rhinos and horned rhinos that were poached both across the whole data set and within populations A and C. Populations B and D could not be tested at the population level due to a lack of dehorning and a lack of poaching cases, respectively.

The effects of dehorning on ICI were tested in females which had given birth to a minimum of two calves prior to dehorning and two calves post-dehorning. If a mother had given birth to more than two calves pre- or post-dehorning, then mean ICI was calculated. As the data were skewed, a paired Wilcoxon test (R base package) was used to compare mean ICI (months) before and after dehorning, paired with female.

Due to limited sample sizes, statistical analysis could not be carried out on causes of death or birth sex ratios before and after dehorning.

## Results

This dataset included information for 265 rhinos. Population A had the longest dataset beginning in 1973, whilst datasets for the other populations were shorter, beginning with the introductions of rhinos to the sites in 2000 (population B), 1996 (population C), and 2008 (population D). A total of 77 individuals across the populations have been dehorned at least once, accounting for 36.8%, 48.2%, and 81.8% of all individuals studied in populations A, C, and D, respectively.

### Female fecundity

**Fig. 1:**
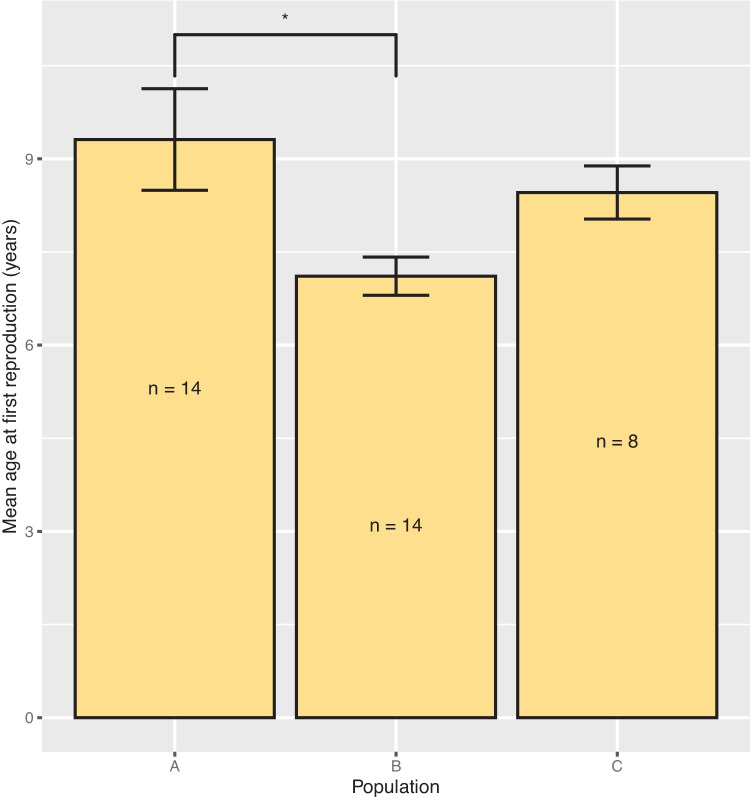
Mean AFR of native black rhino females in populations A (*n* = 14), B (*n* = 14), and C (*n* = 8). No births were recorded in native females in population D. Error bars represent one standard error. (*) represents *p* < 0.05

AFR was calculated for all native females (Fig. 1). Mean AFR was highest in population A (mean = 9.311 years ± 0.818 SE, *n* = 14) and lowest in population B (mean = 7.111 years ± 0.308 SE, *n* = 14). Population was a significant predictor of AFR (LRT = 7.433, *p* = 0.024). However, pairwise comparisons only found a significant difference between populations A and B (*z* = −2.736, *p* = 0.017).

**Fig. 2:**
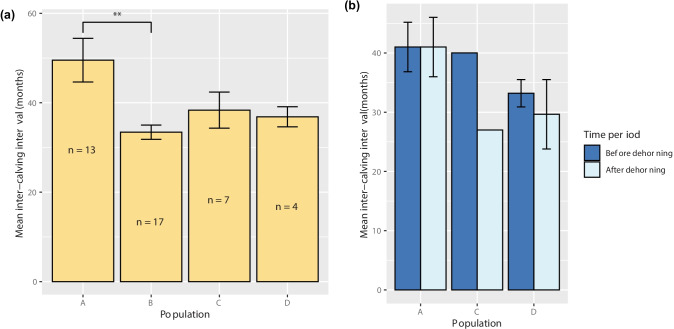
Mean ICI **a** between populations A (*n* = 13), B (*n* = 17), C (*n* = 7), and D (*n* = 4) and **b** before and after females were dehorned in populations A (*n* = 4), C (*n* = 1), and D (*n* = 3). Error bars represent one standard error. (**) represents *p* < 0.01

Mean ICI was calculated for all multiparous females (Fig. 2a). Population A had the longest average ICI (mean = 49.529 months ± 4.882 SE, *n* = 13) and population B had the shortest (mean = 33.415 months ± 1.597 SE, *n* = 17). There was a significant difference in mean ICI between the populations (LRT = 13.281, *p* = 0.004). However, the only significant pairwise comparison was between populations A and B (*z* = −3.705, *p* = 0.001).

ICI was also compared before and after dehorning (Fig. 2b). Population A had a similar average ICI both before (mean = 41.019 months ± 4.179 SE, *n* = 4) and after (mean = 41.014 ± 5.016, *n* = 4) dehorning was carried out. This was the longest mean ICI observed across the populations. The shortest mean ICI was recorded in population C post-dehorning (mean = 26.992 months, *n* = 1). SE could not be calculated for population C as *n* = 1. Mean ICI was shorter after dehorning in all populations, however, this was not significant (*V* = 26, *p* = 0.313).

### Birth sex ratios

**Fig. 3:**
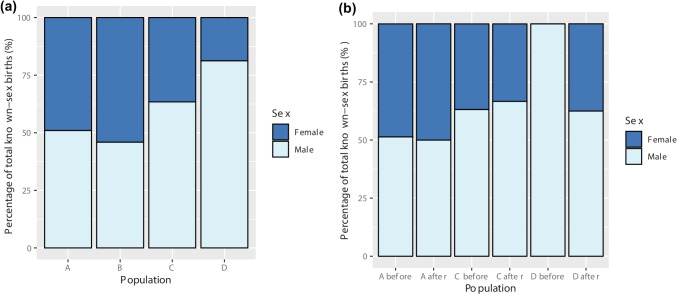
Birth sex ratios of known-sex calves **a** between populations A (*n* = 49), B (*n* = 74), C (*n* = 41), and D (*n* = 16) and **b** before (population A: *n* = 37, population C: *n* = 38, and population D: *n* = 8) and after (population A: *n* = 12, population C: *n* = 3, and population D: *n* = 8) dehorning in each population

Five calves in population A, five in population B, and two in population C were unsexed due either to having died prior to being sexed or being too young to have been sexed at the time of data collection. Mean birth sex ratio was close to 1:1 in populations A (*n* = 49) and B (*n* = 74); however, in populations C (*n* = 41) and D (*n* = 16), males accounted for 63.4 and 81.3% of births, respectively (Fig. 3a). Binomial tests found no significant difference in the number of males compared to females born in populations A, B, or C (Table 2). However, there was a significantly higher proportion of male births in population D (*p* = 0.021, 95% confidence interval (CI) = 0.544, 0.960). Birth sex ratios both before (51.4% male, *n* = 37) and after dehorning (50.0% male, *n* = 12) were close to 1:1 in population A (Fig. 3b). Population C had a slightly higher proportion of male births both before (63.2%, *n* = 38) and after (66.7%, *n* = 3) dehorning. All calves born prior to dehorning in population D (*n* = 8) were male, whereas this decreased to 62.5% of births after dehorning (*n* = 8).

**Table 2:** Results of the binomial tests carried out to compare the ratio of male to female births in each population

**Population**	***P*-value**	**95% confidence intervals**
A	1.000	0.363, 0.656
B	0.561	0.343, 0.579
C	0.117	0.469, 0.779
**D**	**0.021**	**0.544, 0.960**

Bold values highlight statistically significant results

### Calf survival

**Fig. 4:**
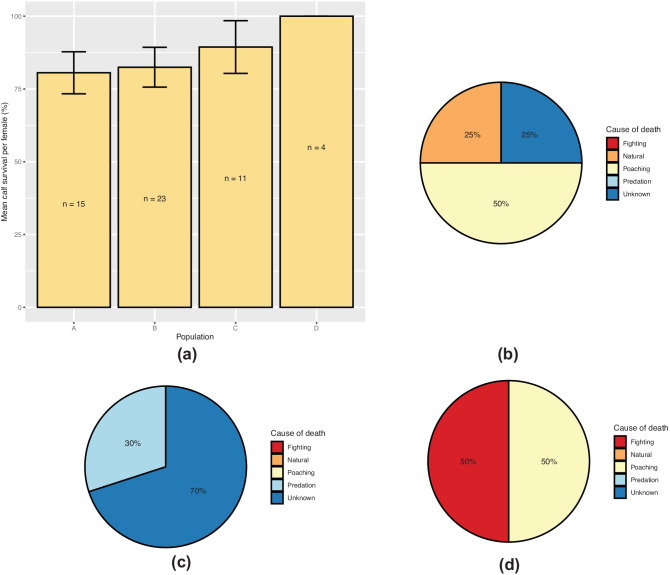
**a** mean calf survival per female in populations A (*n* = 15), B (*n* = 23), C (*n* = 11), and D (*n* = 4), and **b**–**d** cause of calf death as proportions of the total number of calf deaths in **b** population A, **c** population B, and **d** population C. Error bars represent one standard error

Mean calf survival per female was greater than 80.0% in all populations (Fig. 4a), with the lowest survival rate recorded in population A (mean = 80.6% ± 7.213 SE, *n* = 15). Population D had a 100.0% calf survival rate (*n* = 4). There was no significant difference in calf survival between the populations (LRT = 0.658, *p* = 0.883) and there were no significant pairwise comparisons.

Cause of death was assessed for all individuals which did not survive to the sub-adult stage (Fig. 4b–d). Poaching accounted for 50% of calf deaths in populations A and C. Fighting accounted for all other deaths in population C, while the remaining deaths in population A were attributed to natural (25%) and unknown (25%) causes. Predation (30%) and unknown (70%) were the only causes of calf deaths in population B.

**Fig. 5:**
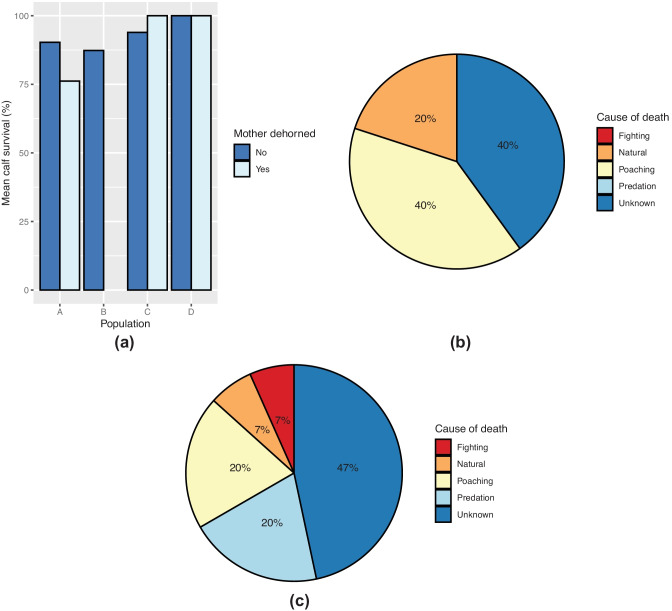
**a** percentage calf survival to the sub-adult stage of the total number of calves born per population between dehorned (*n* = 43) and horned (*n* = 147) mothers and **b**, **c** cause of calf death as percentages of total calf deaths between **b** dehorned (*n* = 5) and **c** horned (*n* = 15) mothers

Calf survival was greater than 75% in all groups when comparing dehorned and horned mothers (Fig. 5a). All calves survived to sub-adults for all mothers in population D and dehorned mothers in population C. Calves born to dehorned mothers in population A had the lowest survival rate (76.1%, *n* = 21). Calf survival was not significantly different between dehorned and horned mothers (LRT = 0.837, *p* = 0.360, *n* = 190). The primary cause of death (Fig. 5b, c) in both groups was unknown (40%). Poaching (40%) and natural causes (20%) resulted in the additional deaths of calves of dehorned mothers. No fighting or predation-related deaths were reported in this group. In calves of horned mothers, poaching and predation each contributed to 20% of deaths, and the remaining proportion were caused equally by fighting and natural deaths.

### Cause of death

**Fig. 6:**
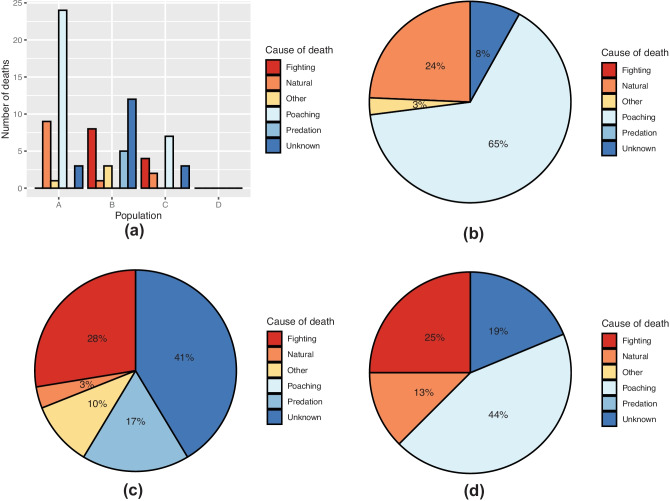
Cause of death across the populations represented as **a** frequencies and **b**–**d** proportions in **b** population A (*n* = 37), **c** population B (*n* = 29), and **d** population C (*n* = 16)

A total of 82 deaths, including both adult and calf deaths, were recorded across the populations from 2001 to 2020 (Fig. 6a–d). Four deaths were categorised as ‘other’, three of which occurred in population B and one in population A. These were attributed to capture-related complications, birth complications, abdominal abscessation and euthanasia following the development of myopathy having become stuck in the mud.

A total of 13 of these deaths occurred in individuals that had been translocated into the populations, while six were individuals that had entered into the populations naturally, and the remaining 63 were individuals that had been born in the populations. Two translocated individuals died within 1 year of introduction to the population. Both of these were translocated into population B and included one male and one female. The female was only 1 year old at the date of translocation. Both deaths were attributed to fighting. All other translocated individuals survived for more than 2 years after introduction.

The largest proportions of deaths in populations A (64.9%, *n* = 37) and C (43.8%, *n* = 16) were caused by poaching, whereas no poaching-related deaths were reported in population B. The largest proportion of deaths in population B was due to unknown causes (41.4%, *n* = 29), with fighting being the second most common cause (27.6%). No deaths from predation were recorded in populations A and C, compared to 17.2% in population B.

**Fig. 7:**
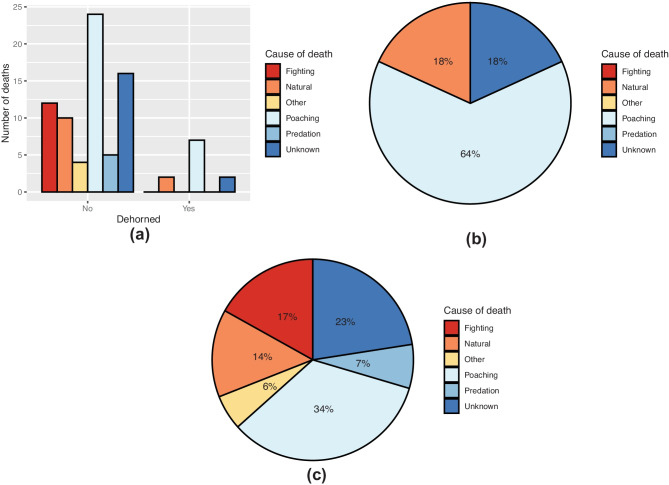
Cause of death between dehorned (*n* = 11) and horned (*n* = 69) individuals represented as **a** frequencies and **b**, **c** proportions of total deaths in **b** dehorned and **c** horned individuals

The cause of death was also compared between dehorned and horned individuals (Fig. 7a–c). Individuals for which it was unknown whether they had ever been dehorned were not included. The largest proportions of deaths in both groups were caused by poaching, accounting for 33.8% of deaths in those individuals who had never been dehorned and 63.6% in those who had. All poaching-related deaths were recorded between 2013 and 2020. The frequency of poaching was three times higher in horned individuals (Table 3), however, dehorned rhinos were not significantly more or less likely to be poached than horned rhinos (*X*^2^ = 0.638, *p* = 0.424, *n* = 265). When assessed at the population level, there was also no significant difference in poaching between dehorned and horned individuals in population A (*X*^2^ = 1.76, *p* = 0.184, *n* = 86) and population C (*X*^2^ = 3.688, *p* = 0.055, *n* = 56).

**Table 3:** Number of poaching incidents across the four study populations

	**Number of rhinos poached**	**Number of rhinos not poached**	**Total**
Dehorned	7	69	76
Horned	24	165	189

Natural and unknown were the only other recorded causes of death in dehorned individuals, whereas deaths from fighting, predation, and other causes were reported in individuals which had never been dehorned, accounting for 16.9%, 7.0%, and 5.6% of deaths, respectively.

### Lifespan

**Fig. 8:**
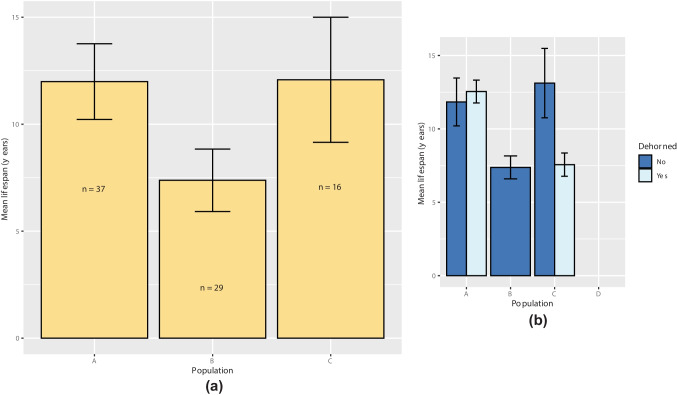
Mean lifespan between **a** populations A (*n* = 37), B (*n* = 29), and C (*n* = 16) and **b** dehorned (*n* = 11) and horned (*n* = 71) individuals. Error bars represent one standard error

Mean lifespan was calculated for all populations (Fig. 8a). No deaths were recorded in population D. Population B had the lowest average lifespan (mean = 7.376 years ± 1.459 SE, *n* = 29). Population A included the oldest recorded individual at 39.96 years old (mean = 11.989 years ± 1.769 SE, *n* = 37), whilst population C had a mean lifespan of 12.078 years ± 2.924 SE, *n* = 16. There was no significant difference in lifespan between the populations (LRT = 4.797, *p* = 0.091), with no significant pairwise comparisons. Lifespan was also compared between dehorned (*n* = 11) and horned (*n* = 71) individuals in each population (Fig. 8b). Dehorning was not a significant predictor of lifespan (Beta = 0.373 ± 0.366, LRT = 1.085, *p* = 0.298). The number of times that an individual had been dehorned also had no significant effect on lifespan (beta = 0.359 ± 0.322, LRT = 1.296, *p* = 0.255).

## Discussion

Dehorning is commonly used as an anti-poaching technique across Africa, however, there are relatively few studies on the impacts of this, particularly in black rhinos. This study aims to build on existing knowledge of the impacts of dehorning on population productivity by comparing AFR, ICI, birth sex ratios, calf survival, lifespan, and cause of death between four Namibian populations of black rhino that have been exposed to varying levels of dehorning.

### Female fecundity

This study identified population differences in both AFR and ICI. Mean AFR in populations B and C, and mean ICI in populations B, C, and D were all as expected for the black rhino based on the current evidence in the literature. Law et al. (2013) reported a mean AFR of 6.66 years with a range up to 9.25 years and a mean ICI of 29 months in a black rhino population in the Great Fish River Reserve, South Africa, while Hrabar and Du Toit (2005) found a mean AFR of 7.25 years with a range of 6–8.82 years and a mean ICI of 33.96 months in Pilanesberg National Park, South Africa. Freeman et al. (2014) reported a mean AFR of 7.35 years with a range of 5.8–9 years in one population of black rhinos and 7.54 years with a range of 7.25–9 years in a second population, both found in Addo Elephant National Park, South Africa. Mean ICI in these two populations was 39.10 months (range 16–51 months) and 27.27 months (range 21–49 months) respectively. In contrast, population A had a longer mean ICI and AFR than these studies, and both were significantly higher than in population B. Populations C and D had similar mean ICIs to B, although these were not significantly different to A, possibly due to smaller sample sizes reducing statistical power. Population A faces the most extreme climatic conditions of the four populations, living in a desert habitat with sparse vegetation cover and the lowest average annual rainfall. Additionally, the average annual rainfall of 150 mm is much lower than the 630 and 452 mm recorded in the populations studied by Hrabar and Du Toit (2005) and Law et al. (2013), respectively, and the 445 mm and 445–600 mm documented in the two populations studied by Freeman et al. (2014). Large terrestrial mammals face greater stresses in arid environments due to high exposure, high temperatures, and low availability of food and water, requiring them to adapt both physiologically and behaviourally. This can include making trade-offs between the need to find shade and reduce energy exposure to prevent overheating, with the need to travel greater distances to find food and water (Fuller et al. 2016). It is, therefore, possible that there is lower calf survival in population A as a result of the arid environment requiring mothers to walk greater distances to gain access to food and water, putting strain on young calves. The free-ranging nature of population A outside of a formally protected area means that this group is monitored less intensively. This may cause births to go unrecorded if the calf dies prior to observation, which could explain the increased ICIs observed in this population despite this study finding no significant difference in calf survival. For the same reasons, there is also an increased possibility of abortions between births going unreported, which would also contribute to an increased observed ICI. Alternatively, delayed reproduction has been reported in some species in harsh environments where foraging conditions are too costly and the energy demands of foraging outweigh the calories gained (Bronson 2009; Tuljapurkar 1990). A 2022 review of the reproductive seasonality of rhinos reported that in both black and white rhinos, peak breeding activity or conception occurred at more optimal times of the year, such as during the wet season when there is fresh grass growth (Radeke-Auer et al. 2022). Additionally, years with lower rainfall and thus poorer environmental conditions were found to correlate with a delay in rutting and decreased intensity of rutting in Iberian red deer (*Cervus elaphus hispanicus*) (Millán et al. 2021). A similar case has been documented in Karoo rats (*Otomys unisulcatus*), where individuals born early in the year had greater food availability and reached reproductive maturity at a younger age than those born later in the year, at which time conditions are more arid and less food is available (Wolhuter et al. 2022). Therefore, it is possible that high AFR and ICI are adaptations of population A to cope with these more harsh climatic conditions.

There was no evidence that dehorning influenced ICI. Firstly, this comes from the finding that there were no significant differences between populations C and D (where dehorning has taken place) and population B (where no dehorning has taken place). Mean ICI was also not significantly different after dehorning, despite being slightly lower in all populations. Similar findings have been reported by Penny et al. (2020a) on the white rhino. It should be noted that female age could not be included in models for ICI due to data limitations, although mean ICI was calculated for each female to reduce the possible impact of female age on ICI. Both Penny’s study and the results presented here feature small sample sizes, and therefore it would be valuable in future studies to use larger sample sizes as well as the inclusion of the age of the mother in models.

Differences in AFR between dehorned and horned individuals could not be tested as dehorning has only taken place in the study populations since 2014, and therefore, no dehorned sub-adult females had given birth to their first calf at the time of the study. Penny et al. (2020a) found that mean AFR was slightly lower after dehorning, although the sample size was low, reducing statistical power. This is an important area for future study as several nulliparous females have now been dehorned in Namibia. AFR is an important factor influencing population productivity, and therefore, monitoring and understanding the impacts of dehorning on AFR is essential to detect any potential adverse effects.

### Birth sex ratios

Population sex ratios are a vital factor in maximising productivity and genetic diversity in any species. There has been some concern that exposure to stressors such as immobilisation and translocation can result in skewed rhino birth sex ratios through sex-differential embryo death. This concern is based on a study which found a significant reversal from a male- to female-biased birth sex ratio between pregnant females that were translocated in the early gestation period and those translocated in the mid-gestation period (Linklater 2007). However, in the current study, birth sex ratios were almost equal when compared within the populations before and after dehorning. Additionally, although population D had a significantly higher proportion of male than female births, this was not observed in populations A and C, which have both also undergone dehorning. This suggests that dehorning is unlikely to have caused the skewed ratios observed in population D. However, due to the low sample size and the fact that statistical analysis could not be carried out to directly compare birth sex ratios before and after dehorning within the same population, this conclusion should be treated with caution, and future studies with larger sample sizes will be important to further investigate this.

Other factors may be responsible for the observed difference in birth sex ratios in population D, such as climatic conditions, population densities, quality/quantity of browse, or chance. The authors have also observed predominantly male offspring in several other custodianship populations (not included in this study) across Namibia prior to the implementation of dehorning (Internal MEFT database, unpublished), further suggesting that additional causes likely play a role. Two studies have reported that years with greater annual rainfall were correlated with a higher male bias in black rhino calves conceived during those years (Hrabar and Du Toit 2005; Berkeley and Linklater 2010), although additional studies with larger samples sizes would be valuable to increase the available information on the potential correlation between rainfall and sex ratios. Due to data limitations, the effect of climatic conditions could not be included in this study, and further research over a longer time frame is needed to assess the possible contribution of rainfall to the male-biased sex ratio observed in population D. There is hence no evidence to suggest that dehorning influences birth sex ratios.

### Calf survival

A previous study on black rhinos in Namibia claimed a 100% mortality rate due to predation in the calves of dehorned mothers (Berger and Cunningham 1994). In contrast, this study observed a mean survival of greater than 75% (i.e. a mortality rate of less than 25%). The study by Berger and Cunningham has since been highly criticised due to: a small sample size (*n* = 3), a lack of explanation as to how predator abundance and density were measured, the study coinciding with a period of severe drought in Namibia (Lindeque and Erb 1995), and a lack of direct evidence for death (no carcasses were reported) or predation. Other groups have since reported that they have observed no difference in calf survival between dehorned and horned mothers (Atkinson 1996; Du Toit and Anderson 2013). The reports of Atkinson (1996) and Du Toit and Anderson (2013), combined with the fact that the only population for which any calf deaths were attributed to predation in the present study was population B, in which dehorning has never taken place, suggest that the observed overall survival rate of over 75% in this study is likely a more accurate estimate than the conclusions made by Berger and Cunningham.

It is likely that other factors have a greater influence on calf survival than dehorning, such as predator and prey abundance and experience of the mother. Predator abundance varied between the populations, with lions, spotted and brown hyena, leopard and cheetah present in populations A and B, no large predators in population C, and spotted hyena and leopard in D. Predation of black rhino calves by both spotted hyena and lions has been reported previously (Fyumagwa and Nyahongo 2010; Mills et al. 2006; Sillero-Zubiri and Gottelli 1991; Hitchins and Anderson 1983; le Roex and Ferreira 2020; Plotz and Linklater 2009). However, due to data limitations, predator type and density could not be included in the models in this study, meaning that no conclusions can be made about the effects of predator abundance and density on rhino predation in this study. It is also possible that age of the mother, and therefore, experience may influence calf survival. However, this could not be included in the models in this study due to data limitations. Therefore, an important future study would be to use a larger dataset including additional rhino populations and incorporate predator abundances and age of the mother into the models to better assess the potential influence of dehorning on susceptibility to predation, particularly as although both populations A and B had similar large predators present, no predation was recorded in population A while three cases were recorded in population B. It should be noted that frequently, when rhino calves go missing, the cause of death is unknown and could possibly be due to predation. However, this would require closer monitoring of mothers and calves, which is often not possible. Although future studies assessing predator densities are required to add support to this, the results presented here are in line with observations of experienced rhino conservationists who report that they have observed no difference in calf mortality between dehorned and horned mothers (Atkinson 1996; Du Toit and Anderson 2013). Therefore, there is currently little or no evidence to suggest that dehorning directly impacts calf survival.

### Cause of death

No fighting-related deaths were reported in dehorned individuals in comparison to 12 in horned individuals. In addition, fighting was also the most frequent known cause of death in population B where no individuals have been dehorned. While this might reflect that dehorning of even a proportion of the population may reduce deaths from fighting, there may be other factors underlying this result such as variation in rhino densities which may be critical in the likelihood of fighting. It is also possible that dehorning may reduce the incidence of fighting, as has been reported in the Ziwa Rhino Sanctuary, Uganda (Patton et al. 2018). However, this was not possible to examine this here. Additionally, it is possible that dehorning of only a proportion of a population could place dehorned individuals at a disadvantage in conflicts and that this was not reflected in the study due to small sample sizes. These potential disadvantages could present as nonfatal impacts, such as through reduced access to mates or territory. Further studies with larger sample sizes are needed to assess this as other factors are also likely to be important, such as population densities. The impact of dehorning on fatalities from fighting is an important consideration, particularly in small managed populations with high densities where males can be more aggressive, resulting in increased fighting and thus elevating the risk of fatalities (Patton et al. 2018). Therefore, it is possible that dehorning in these cases may be beneficial in reducing fatalities even if poaching is not a problem in the area. Further investigation into the effectiveness of dehorning in preventing fatalities from fighting would be valuable to better inform whether dehorning is a cost-effective approach to increasing rhino population sizes.

The fact that so few deaths were recorded in other categories explains why poaching contributed to a larger proportion of deaths in dehorned individuals. No deaths due to predation were recorded in dehorned individuals, and hence there is no evidence here that overall susceptibility to predators, in addition to calf susceptibility, is increased by dehorning, although due to the limited sample size, further study is needed to confirm this.

Of note, the finding that only two rhinos died within a year of translocation into the populations whilst all other translocated individuals survived for a minimum of 2 years post-translocation is important as translocation is frequently used by conservationists. It is therefore reassuring that in this study, almost all translocated individuals survived for over 2 years.

The observation that poaching was the most prevalent cause of death in dehorned individuals is counter-intuitive as dehorning is used as an anti-poaching method. However, reports have shown that dehorning without other anti-poaching strategies is insufficient to prevent poaching (Lindsey and Taylor 2011). Poaching contributed to the greatest proportion of deaths in dehorned individuals, however, there was no significant difference in the poaching rate between dehorned and horned individuals. Although it is possible therefore that dehorning is ineffective at preventing poaching in these populations, it should be considered that dehorning usually takes place in areas with historically high poaching. Therefore, individuals living in these populations may already be at higher risk of poaching due to factors such as proximity to main roads, international borders, and other security measures (Lindsey and Taylor 2011). Incidence of poaching is likely to be a cost–benefit situation, influenced not only by the reward to the poacher (through the value of horn obtained) but also the risk of being caught and the effort required to poach (Du Toit and Anderson 2013). Therefore, if the value of horn remaining after dehorning is considered by the poacher to outweighs the risks of being caught or the effort involved in poaching, it is possible that dehorned rhinos will still be targeted (Lindsey and Taylor 2011). All poaching deaths occurred between 2013 and 2020, coinciding with some of the highest annual reports of poaching in Africa and some of the highest reported prices paid to poachers (UNODC 2020; Emslie et al. 2019). It is possible therefore that the poaching of dehorned individuals in this study was a result of the high value of rhino horn at the time, making even the small portion of horn remaining after dehorning valuable and altering the cost–benefit balance, encouraging poachers to venture into areas with greater security due to the increased value of potential reward. Dehorning is often used as a part of a series of other measures in an attempt to deter poachers, making it difficult to quantify the contribution of dehorning to reducing poaching. As dehorning was only restarted in Namibia in 2014, it is also possible that poachers were unaware of this and therefore targeted dehorned rhinos thinking that they were horned. Further research to control for confounding factors, such as variation in poaching pressure between different areas, is required to assess the effectiveness of dehorning as an anti-poaching tool. This was beyond the scope of this study, which was to examine whether there is any evidence to suggest that population productivity is negatively affected by dehorning. The cause of death results in this study should be treated with caution due to the small sample sizes preventing statistical analysis, however, it is reassuring that dehorned individuals did not appear to be more at risk of death from fighting, poaching, or predation than horned individuals.

### Lifespan

Maximising lifespan is vital for population growth in rhino populations as age is correlated with reproductive output in females (Cain et al. 2014). Interestingly, the lowest mean lifespan was recorded in population B, in which no dehorning occurred. Despite being more than 4 years shorter than the next lowest mean lifespan, this result was not significant. It was also found that the number of times an individual was dehorned had no significant effect on lifespan. Although it may be the case that dehorning has no effect on lifespan, it is possible that dehorning has subtle effects on lifespan but that these were not detected in this study due to the low sample size and the short study period. There was also a larger margin for error for birth dates of introduced compared to native individuals, which reduced statistical power. The low lifespan in population B may be explained by the possibility that dehorning might be protective against fighting-related deaths (“Cause of death” section), however, further studies are required to test this. Consequently, no evidence was found here to suggest that lifespan is greater (or lower) in dehorned individuals.

### Study limitations

There were firstly several analyses that could not be conducted due to insufficient data, such as lack of information about the sex or cause of death of some calves. For example, it was not possible to assess whether differences in birth sex ratios before and after dehorning or in the cause of death both between the populations and between dehorned and horned individuals were significant due to the lower sample size. Moreover, the data was limited to four populations in Namibia. In future, it will be important to assess whether the results observed are also found in other populations in other countries.

Similarly, the study period was too short to allow a complete understanding of the impacts of dehorning. Specifically, it was not possible to assess the impact of dehorning on AFR as none of the dehorned females had yet produced offspring. Additionally, population D was only established in 2008 and no natural deaths have been recorded in the population, meaning that lifespan and cause of death could not be assessed. It would be valuable for a follow-up study to be carried out in the future once this data is available.

It is also possible that the translocation of individuals could influence both cause of death and lifespan. However, as 11 of the 13 translocated individuals survived for over 2 years after translocation into the populations, suggesting that translocation did not have an immediate effect on mortality, it was decided to include these individuals in analyses. Further research on the impact of translocation would be valuable, and caution should be taken when interpreting the results of the analyses of lifespan and cause of death outlined here. Additionally, lack of information on individuals prior to introduction to the populations meant that this could not be taken into consideration in this study. In future it would be valuable to obtain records from the source population if possible, as conditions prior to introduction may influence variables such as lifespan and cause of death.

There are also a number of factors likely to influence the variables assessed in this study. For example, environmental conditions such as rainfall, predator type and abundance, rhino densities or age of the mother in the analysis of calf survival or ICI. Sufficient data was not available on these factors to fully account for them in analyses. To attempt to account for some of these factors, the effects of the population were controlled for statistically, given that environmental conditions may differ considerably between populations. However, it will be highly important to attempt to account for each of these factors in future studies so that more solid and robust conclusions about dehorning can be made.

To maximise statistical power and avoid issues associated with multiple testing, data from all populations were aggregated prior to analysis. To control for potential differences between populations, especially given the variation in the timeframe, ‘population’ was included as a covariate in all GLMs.

## Conclusion

No statistically significant effects of dehorning on population productivity in the black rhino were identified across a range of measures, including AFR, ICI, birth sex ratios, calf survival, or lifespan. The lack of significant result may stem from study limitations such as a small sample size. However, the results are in agreement with several recent studies which also did not find any long-term negative effects of dehorning in black or white rhinos (Du Toit and Anderson 2013; Badenhorst et al. 2016; Penny et al. 2020a, b). The balance of the evidence hence suggests that dehorning has little or no observable impact on population productivity. Black rhinos are closely monitored due to their critically endangered status and therefore there is already a lot of data available on this species, however this is often highly confidential due to the risks of sharing data and it falling into the hands of poachers. One of the most important future studies will be to collate the already available data from as many different sources as possible across multiple countries to carry out a wider analysis on the effects of dehorning. This will also allow for factors such as annual changes in climatic conditions, large predator abundances, rhino densities, and female age to be better accounted for. Similarly, investigation into the impacts of dehorning on AFR are particularly important due to the number of nulliparous females which have now been dehorned in Namibia.

To date, studies on the effectiveness of dehorning as an anti-poaching deterrent have been limited. This study found no significant difference in poaching rate between dehorned and horned individuals across the four populations studied. While this may again simply reflect insufficient power to detect an effect or other study limitations, it could also cautiously be interpreted as indicating a negligible impact of dehorning as an anti-poaching deterrent. Dehorning is an expensive technique, and therefore, it is vital that further studies are carried out across Namibia and between Namibia and other countries, to assess this on a larger scale in an attempt to quantify whether dehorning is effective at deterring poaching and is cost-efficient. Again, it would be useful for future studies to collate datasets already available from as many sources as possible, across different countries, to investigate the effectiveness of dehorning. Consideration should also be given to the effects of dehorning one population on neighbouring populations where dehorning has not occurred, as there is a possibility that this will increase poaching pressure in other areas. The impact of dehorning on rhino behaviour should also be considered, as it is possible that dehorning activities could make rhinos more fearful of humans, making them more difficult to monitor for overall health, potentially resulting in additional mortalities.

In sum, while it is reassuring that no negative impacts of dehorning on rhino population productivity were identified, this is by no means conclusive, and much more research is needed. It will also be important to conduct further research to investigate the effectiveness of dehorning as an anti-poaching deterrent.

## References

[CR1] Alibhai SK, Jewell ZC, Towindo SS (2001). Effects of immobilization on fertility in female black rhino (*Diceros bicornis*). J Zool.

[CR2] Atkinson MW (1996) Dehorning. IRF Special Feature. Available at: http://www.rhinoresourcecenter.com/pdf_files/127/1273617336.pdf. Accessed 6 Jan 2021

[CR3] Atkinson MW, du Toit R, Radcliffe RW, Dooley JL, Kock MD (2002). Response to Alibhai, Jewell and Towindo. J Zool.

[CR4] Badenhorst M, Ganswindt A, Otto M, Van der Goot AC (2016). Stress steroid levels and the short-term impact of routine dehorning in female southern white rhinoceroses (Ceratotherium simum simum). African Zoology.

[CR5] Berger J, Cunningham C (1994). Phenotypic alterations, evolutionarily significant structures, and rhino conservation. Conserv Biol.

[CR6] Berkeley EV, Linklater WL (2010) Annual and seasonal rainfall may influence progeny sex ratio in the black rhinoceros. South African J Wildlife Res 24-month Delayed Open Access 40(1):53–57

[CR7] Brodie JF, Muntifering J, Hearn M, Loutit B, Loutit R, Brell B, Uri-Khob S, Leader-Williams N, Du Preez P (2011). Population recovery of black rhinoceros in north-west Namibia following poaching. Anim Conserv.

[CR8] Bronson FH (2009). Climate change and seasonal reproduction in mammals. Philosophical Transactions of the Royal Society B: Biological Sciences.

[CR9] Cain B, Wandera AB, Shawcross SG, Edwin Harris W, Stevens-Wood B, Kemp SJ, Okita-Ouma B, Watts PC (2014). Sex-biased inbreeding effects on reproductive success and home range size of the critically endangered black rhinoceros. Conserv Biol.

[CR10] Cheteni P (2014). An analysis of antipoaching techniques in Africa: a case of rhino poaching. Environmental Economics.

[CR11] Crookes DJ, Blignaut JN (2019) In approach to determine the extinction risk of exploited populations. J Nat Conserv 52

[CR12] Du Toit R (2001). Rationale for ongoing radio-collaring of Black Rhinos–a response to Alibhai and Jewell. Oryx.

[CR13] Du Toit R, Anderson N (2013) Dehorning rhinos. Wildlife Ranching, 2013 Autumn 82–85

[CR14] Emslie R (2020a) Ceratotherium simum. The IUCN Red List of Threatened Species. Available at: https://www.iucnredlist.org/species/4185/45813880. Accessed 15 Aug 2022

[CR15] Emslie R, Milliken T, Talukdar B, Burgess G, Adcock K, Balfour D, Knight MH (2019) 'African and Asian Rhinoceroses – status, conservation and trade: a report from the IUCN Species Survival Commission (IUCN SSC) African and Asian Rhino Specialist Groups and TRAFFIC to the CITES Secretariat pursuant to Resolution Conf. 9.14 (Rev. CoP17) ', CITES 18th Meeting of the Conference of Parties, Geneva, Switzerland, 17–28 August: CITES Secretariat

[CR16] Emslie RH (2020b) Diceros bicornis. The IUCN Redlist of Threatened Species. Available at: 10.2305/IUCN.UK.2020b-1.RLTS.T6557A152728945.en. Accessed 6 Jan 2021

[CR17] Emslie RH, Adcock K, Hansen HB (1995) Fine tuning the rhino management group age class system

[CR18] Millán F, M., Carranza, J., Perez-Gonzalez, J., Valencia, J., Torres-Porras, J., Seoane, J. M., de la Peña, E., Alarcos, S., Sánchez-Prieto, C. B. and Castillo, L.  (2021). Rainfall decrease and red deer rutting behaviour: weaker and delayed rutting activity though higher opportunity for sexual selection. PLoS ONE.

[CR19] Ferreira SM, Greaver C, Knight GA, Knight MH, Smit IPJ, Pienaar D (2015). Disruption of rhino demography by poachers may lead to population declines in Kruger National Park, South Africa. PLoS ONE.

[CR20] Freeman EW, Meyer JM, Bird J, Adendorff J, Schulte BA, Santymire RM (2014) Impacts of environmental pressures on the reproductive physiology of subpopulations of black rhinoceros ( Diceros bicornis bicornis ) in Addo Elephant National Park, South Africa, Conserv Physiol 1(2) 10.1093/conphys/cot034 PMC4732468 27293618

[CR21] Fuller A, Mitchell D, Maloney SK, Hetem RS (2016) 'Towards a mechanistic understanding of the responses of large terrestrial mammals to heat and aridity associated with climate change'. Clim Change Resp 3(10)

[CR22] Fyumagwa R, Nyahongo J (2010). Black rhino conservation in Tanzania: translocation efforts and further challenges. Pachyderm.

[CR23] Gasaway WC, Gasaway KT, Berry HH (1996). Persistent low densities of plains ungulates in Etosha National Park, Namibia: testing the food-regulating hypothesis. Can J Zool.

[CR24] Haas TC, Ferreira SM (2016). Combating rhino horn trafficking: the need to disrupt criminal networks. PLoS ONE.

[CR25] Hitchins PM, Anderson JL (1983) 'Reproduction, population, characteristics and management of the black rhinoceros Diceros bicornis minor in the Hluhluwe/Corridor/Umfolozi Game Reserve Complex'. South African J Wildlife Research-24-month Delayed Open Access 13(3):78–85

[CR26] Hothorn T, Bretz F, Westfall P (2008). Simultaneous inference in general parametric models. Biom J.

[CR27] Hrabar H, Du Toit JT (2005). Dynamics of a protected black rhino (Diceros bicornis) population: Pilanesberg National Park, South Africa. Anim Conserv.

[CR28] Inman EN, Hobbs RJ, Tsvuura Z, Valentine L (2020). Current vegetation structure and composition of woody species in community-derived categories of land degradation in a semiarid rangeland in Kunene region, Namibia. Land Degrad Dev.

[CR29] Kock MD, Atkinson M (1993) Report on dehorning of Black (Diceros bicornis) and White (Ceratotherium simum) rhinoceroses in Zimbabwe, Harare, Zimbabwe: Department of National Parks and Wild Life Management

[CR30] Law PR, Fike B, Lent PC (2013). Mortality and female fecundity in an expanding black rhinoceros (Diceros bicornis minor) population. Eur J Wildl Res.

[CR31] le Roex N, Ferreira SM (2020). Age structure changes indicate direct and indirect population impacts in illegally harvested black rhino. PLoS ONE.

[CR32] Lemieux A, Corné E, Lemieux AM (2014). Rhino poaching in Kruger National Park, South Africa: aligning analysis, technology and prevention. Situational prevention of poaching.

[CR33] Lindeque M, Erb KP (1995). Research on the effects of temporary horn removal on black rhinos in Namibia. Pachyderm.

[CR34] Lindsey PA, Taylor A (2011) A study on the dehorning of African rhinoceroses as a tool to reduce the risk of poaching, Johannesburg: endangered wildlife trust

[CR35] Linklater WL (2007). Translocation reverses birth sex ratio bias depending on its timing during gestation: evidence for the action of two sex-allocation mechanisms. Reprod Fertil Dev.

[CR36] Matson T, Putland D, Jarman P, le Roux J, Goldizen A (2006). Influences of parturition on home range and microhabitat use of female black-faced impalas. J Zool.

[CR37] Mendelsohn JM, El Obeid S (2002) The communal lands in Eastern Namibia. Windhoek, Namibia: Raison

[CR38] Mendelson J, Jarvis A, Roberts C, Robertson T (2002). Atlas of Namibia: a portrait of the land and its people.

[CR39] Milliken T, Shaw J (2012) The South Africa–Viet Nam Rhino Horn Trade Nexus: a deadly combination of institutional lapses, corrupt wildlife industry professionals and Asian crime syndicates, Johannesburg, South Africa.

[CR40] Mills A, Morkel P, Amiyo A, Runyoro V, Borner M, Thirgood S (2006). Managing small populations in practice: black rhino Diceros bicornis michaeli in the Ngorongoro Crater, Tanzania. Oryx.

[CR41] Mukwazvure A, Magadza TB (2014). A survey on anti-poaching strategies. International Journal of Science and Research.

[CR42] Namibian Ministry of Environment and Tourism (2010) Namibia’s Draft Fourth National Report to the United Nations Convention on Biological Diversity (UNCBD). Available at: https://www.cbd.int/doc/world/na/na-nr-04-en.doc. Accessed 15 Aug 2022

[CR43] Patton F, Campbell P, Genade A (2018). The effect of dehorning adult male rhinos on the frequency of fighting at Ziwa Rhino Sanctuary. Pachyderm.

[CR44] Penny SG, White RL, MacTavish D, MacTavish L, Scott DM, Pernetta AD (2020). Does dehorning lead to a change in inter-calf intervals in free-ranging white rhinoceros?. Pachyderm.

[CR45] Penny SG, White RL, MacTavish L, Scott DM, Pernetta AP (2020b) 'Negligible hormonal response following dehorning in free-ranging white rhinoceros (Ceratotherium simum)'. Conserv Physiol 8(1):coaa117 10.1093/conphys/coaa117 PMC7771576 33408864

[CR46] Plotz RD, Linklater WL (2009). Black rhinoceros (Diceros bicornis) calf succumbs after lion predation attempt: implications for conservation management. African Zoology.

[CR47] Radeke-Auer K, Wittwer A, Aust J, Roller M, Müller D, von Houwald F, Steck B, Biddle R, Versteege L, Clauss M (2022). Reproductive non-seasonality in rhinoceroses: a review of the in-situ literature and birth records of ex-situ institutions. Journal of Zoo and Aquarium Research.

[CR48] RStudio Team (2021) RStudio: Integrated Development Environment for R. RStudio, PBC, Boston, MA

[CR49] Sillero-Zubiri C, Gottelli D (1991). Threats to Aberdare rhinos: predation versus poaching. Pachyderm.

[CR50] Truong VD, Dang NVH, Hall CM (2016). The marketplace management of illegal elixirs: illicit consumption of rhino horn. Consum Mark Cult.

[CR51] Tuljapurkar S (1990). Delayed reproduction and fitness in variable environments. Proc Natl Acad Sci.

[CR52] t’ Sas-Rolfes, M.  (2016). Rhino poaching: what is the solution. The Solutions Journal.

[CR53] UNODC (2020) World Wildlife Crime Report 2020: Trafficking in protected species.

[CR54] Wolhuter L, Thomson J, Schradin C, Pillay N (2022). Life history traits of free-living bush Karoo rats (Otomys unisulcatus) in the semi-arid Succulent Karoo. Mammal Research.

